# Prediction and Prevention in the Clinical High-Risk for Psychosis Paradigm: A Review of the Current Status and Recommendations for Future Directions of Inquiry

**DOI:** 10.3389/fpsyt.2021.770774

**Published:** 2021-10-22

**Authors:** Michelle A. Worthington, Tyrone D. Cannon

**Affiliations:** ^1^Department of Psychology, Yale University, New Haven, CT, United States; ^2^Department of Psychiatry, Yale University, New Haven, CT, United States

**Keywords:** clinical high-risk, psychosis, risk prediction, prevention, review, schizophrenia

## Abstract

Prediction and prevention of negative clinical and functional outcomes represent the two primary objectives of research conducted within the clinical high-risk for psychosis (CHR-P) paradigm. Several multivariable “risk calculator” models have been developed to predict the likelihood of developing psychosis, although these models have not been translated to clinical use. Overall, less progress has been made in developing effective interventions. In this paper, we review the existing literature on both prediction and prevention in the CHR-P paradigm and, primarily, outline ways in which expanding and combining these paths of inquiry could lead to a greater improvement in individual outcomes for those most at risk.

## Introduction

The ability to detect, predict, and delay or prevent the onset of psychotic disorders has been the focus of the clinical high-risk for psychosis (CHR-P) research paradigm for the past 25 years (1). Approximately 20–25% of at-risk individuals develop psychosis within a 2–3-year study period. The prospective nature of the CHR-P paradigm (2) is conducive to building prognostic models (3) to enable more accurate prediction of psychosis (4, 5). A set of such predictive algorithms is now available (6–10), but none has thus far been widely implemented clinically (11). Significantly less progress has been made in developing interventions that delay or prevent psychosis transition (12) and in tailoring treatment selection based on individual risk profiles. Here, we critically review the status of research on prediction of outcomes and prevention of psychosis. We argue that a better understanding of heterogeneous outcomes of the at-risk state could improve efforts to develop effective and personalized interventions (13). We outline concrete directions for this new scope of inquiry, including incorporating prognostic stratification into intervention studies, considering an array of clinical outcomes (e.g., remission, social and functioning outcomes, and treatment outcomes), validating these outcomes with clinical and biological markers, and implementing state-of-the-art methods within these respective lines of research toward the goal of understanding, predicting, and intervening at the level of the individual.

## Predicting Clinical Outcomes

### Prediction of Psychosis Transition

Most of the work within the CHR-P paradigm to date has focused on building models to predict and individual's likelihood of transition to psychosis. A handful of externally-validated multivariable prediction algorithms—or “risk calculators”—are now available (11, 14). These models, largely comprised of clinical, demographic, and neurocognitive measures, have the potential for assessment in a single outpatient clinic visit.

In the first iteration of the North American Prodrome Longitudinal Study (NAPLS) (6), an empirically derived model identified additional risk factors beyond the CHR syndrome criteria to predict psychosis transition, including genetic risk for schizophrenia with recent functional decline, unusual thought content, and either suspiciousness and paranoia or impaired social functioning. These additional factors increased the positive predictive power from 35 to 74–81% (4). In NAPLS2, predictor variables were selected from the literature for inclusion in an individualized risk calculator for determining risk of psychosis transition (15). These variables included several from the NAPLS1 model plus verbal learning and memory performance, speed of processing, age, stressful life events, and history of trauma. This risk calculator ultimately performed well, achieving a Harrel's C-index of 0.71 (15). Importantly, this model was externally validated in the Early Detection, Intervention, and Prevention of Psychosis Program (EDIPPP) (16) sample (C-index = 0.79) (17) and was later also later validated in the Shanghai at Risk for Psychosis study (SHARP) (10) sample (Area Under the Curve [AUC] = 0.63) (18). This latter validation step showed that while this risk calculator model has potential for eventual broad use in samples outside of the U.S., there may still be significant differences in, for example, a Chinese population which consists of different social and cultural backgrounds that are potentially not captured by a model derived in an American sample.

Outside of the U.S., the Melbourne-based Personal Assessment and Crisis Evaluation (PACE) study (9) developed a classification algorithm based on symptom information (accuracy = 64.6%) (19), which has not been externally validated in an independent sample. Although robust internal cross-validation techniques were used to test generalizability, this approach does not provide sufficient evidence for generalizability given that the model learns iteratively from all of the data. The Shanghai-based SHARP study identified individual SIPS items to include in a prediction model (SIPS-RC). These items overlapped with previous models and included functional decline, unusual thought content, suspiciousness, social anhedonia, expression of emotion, ideational richness and dysphoric mood (AUC = 0.80) (20). When externally validated alongside the NAPLS2 risk calculator in a small (*n* = 68) North American SIPS-based CHR sample, the SIPS-RC and NAPLS2 risk calculator performed with fair and moderate accuracy, respectively, in discriminating between converters and non-converters (SIPS-RC AUC = 0.65, NAPLS2 risk calculator AUC = 0.71) (21). For a more detailed description of each of these predictive algorithms, please refer to Worthington, Cao and Cannon (14).

While external validation has shown promising initial results for these risk algorithms, performance in primary care settings or secondary mental health care settings (e.g., outside of highly specialized CHR-P samples) has yet to be thoroughly tested. A recent study evaluated the performance of the NAPLS2 risk calculator in the European-based Personalized Prognostic Tools for Early Psychosis Management study (PRONIA, www.pronia.eu), which includes individuals experiencing recent-onset depression (ROD) in addition to psychosis risk syndromes (22). After significant calibration across samples to account for cross-consortium differences in demographics and symptom severity, the original NAPLS2 model performed best when validated in the broader risk sample (e.g., CHR-P/ROD), demonstrating the potential prognostic utility of psychosis risk signatures in broader risk populations.

Identifying psychosis in non-risk enriched mental health care settings requires the consideration of comorbid diagnoses from more widely-used instruments such as the Diagnostic and Statistical Manual of Mental Disorders, Fifth Edition (DSM-5) (23) or the International Statistical Classification of Diseases and Related Health Problems, Tenth Revision (ICD-10) (24–26). One such risk calculator was developed using a sample from the South London and the Maudsley (SLaM) National Health Service Foundation Trust services (n = 91,199). Psychosis risk syndrome criteria were necessary but insufficient in predicting the development of psychotic disorders in this secondary mental health care setting. Beyond the CHR-P criteria, ICD-10 diagnoses of acute and transient psychotic disorders and bipolar disorder predicted psychosis onset with fairly high accuracy when externally validated [C-index = 0.79 (27) and C-index = 0.73 (28)]. When validated in a comparable US-based sample (*n* > 2,000,000), the model maintained a good level of accuracy in detecting eventual psychosis onset (C-index = 0.67) (29). A significant challenge to this specific replication included differences in help-seeking behaviors and recruitment strategies between the UK-based discovery sample and the US-based replication sample. Accounting for differences in healthcare culture and economics will be discussed more in depth in a latter portion of this paper.

### Prediction of Functional Outcomes

Levels of global, social and role functioning are consistently evaluated alongside attenuated psychotic symptoms (APS) as ecological markers of personal and occupational impairment. However, level of functioning is infrequently considered as a primary outcome of interest, potentially due to the lack of diagnostic outcome associated with functioning as compared to transition to psychotic illness, which represents a putative disorder. Further, studies vary in the subtype of functioning that is assessed—global functioning as measured by the one-item Global Assessment of Functioning (GAF) scale (30), and social or role functioning as measured by the Global Functioning Social scale (GFS), the Global Functioning Role scale (GFR) (31), and the Social and Occupational Functioning Assessment Scale (SOFAS) (32)—and often focus on the level of functioning that is most impacted in a given sample, which may also potentially dilute the strength and consistency of findings regarding “functioning” in the CHR-P syndrome.

While functioning may improve over time for non-converters, this group often remains impaired as compared to nonpsychiatric healthy controls (33). Among non-converters, remitters have shown improved social functioning, but role functioning remained impaired for all CHR groups as compared to healthy controls (34). Non-remitting non-converters (e.g., “sustainers”) continued to show significant functioning impairments at 6 years of follow-up, suggesting that functioning deficits in this population are enduring and may require specialized interventions (35). Although several “risk calculators” exist to predict psychosis transition, only two comparable multivariable models have been developed to predict functioning outcomes, despite potentially enduring functioning deficits exhibited in non-converters that may require specialized interventions (34, 35). One limitation of developing such models is that it is necessary to either predict functioning on a continuous scale, which may not translate to clear clinical decisions, or to determine cutoffs signifying positive or negative outcomes, which collapses much of the nuance captured in the continuous scales. Nevertheless, it remains important to understand an individual's likelihood for achieving positive or negative functional outcomes independent of the progression of APS.

The PRONIA study team developed accurate and generalizable models to predict social and role functioning at 1 year of follow-up (36). A model that incorporated both clinical and structural neuroimaging data outperformed clinical raters' estimations of social functional outcomes (combined model AUC = 0.86; clinical rater AUC = 0.72). Models predicting role functioning, however, did not outperform clinical raters' estimations. Role functioning may be more sensitive to temporal fluctuations of environmental and clinical factors, creating an additional challenge in producing reliable predictive models of this particular outcome (36, 37). The PACE study team also developed a model predicting functioning, which performed similarly to their model predicting psychosis transition (accuracy = 62.5%) (19). In this model, attention disturbances, asocial anhedonia and thought disorder were most predictive of negative functional outcomes. It is worth noting that neither of these algorithms have yet been replicated in external samples.

### Prediction of Remission Outcomes

Remission from a CHR-P syndrome is an important and relatively unexplored outcome. In NAPLS2, trajectory modeling of non-converters showed that a third remitted in symptoms and functioning (38). Predicting CHR-P remission at the level of the individual could inform treatment decisions by allocating more intensive treatments to those most likely to experience negative outcomes than for those more likely to naturally recover (“remitters”). Understanding protective factors associated with remission could also help to inform and tailor interventions for the CHR-P population as remission may serve as a more proximal treatment outcome for intervention studies as compared to psychosis prevention. Further, remitters are a confound in randomized clinical trials (RCTs) wherein the true efficacy of an intervention may not be detected if a significant portion of participants naturally remit, potentially contributing to an inflated placebo effect (39).

While criteria for both symptom and functional remission have been established (35, 40), the clinical validity associated with the timing and duration of remission has not been adequately studied. An individual may meet remission criteria at only a single study visit and relapse at the next or may remain remitted throughout the remainder of the study—both would be considered “remitters” even though the actual clinical picture may vary considerably. This lack of clarity in remission outcome timing contributes to discrepancies in understanding recovery rates. Between 24 and 51% of non-converters may exhibit symptom remission during follow-up while only 24–28% demonstrated full clinical and functional recovery (33, 34, 41, 42). It is also unknown whether remission represents a risk factor for later relapse of APS (or even eventual psychosis transition)—an outcome that can only be studied with very long follow-up periods.

Individual clinical predictors of remission may include higher neurocognitive functioning at baseline, higher levels of social and/or role functioning at baseline, and lower levels of APS at baseline (34, 43, 44). Only one study has developed a generalizable multivariable model predicting remission as an outcome variable. This study took a data-driven approach to selecting relevant features and building a model predicting remission in the NAPLS3 sample and performed well when tested in an independent validation sample (AUC = 0.66), exhibiting performance comparable to models predicting conversion to psychosis (45).

### Gaps and Future Directions in Prediction

Although progress has been made in developing generalizable risk models for multiple outcomes from the CHR-P syndrome, there is still room for improvement before prediction algorithms enter the clinic. Thorough external validation strategies have been limited to date. External testing in diverse risk cohorts and varied health care settings is necessary to: (1) account for cross-site and cross-country sample differences; (2) ensure compatibility across clinical instruments; (3) test the risk signatures measured by each algorithm; and (4) ensure the specificity of eventual psychosis onset as compared to negative outcomes more broadly (26, 46, 47). Methodological differences in both feature selection (e.g., data-driven and theory-driven) and model development (e.g., Cox regression and machine learning classifiers) provide novel avenues of model discovery but limit the possibility of direct comparisons of performance between models developed in heterogeneous samples (11).

Levels of patient distress and pathways of ascertainment differ across cohorts and pose an additional challenge to the development of prediction models. The studies described here rely on either intensive outreach to the general population (self-referral), relationships with established healthcare providers (clinician-referral), or a combination thereof. These approaches yield samples with different levels of baseline conversion risk. Self-referrals result in a less enriched risk sample and a lower eventual rate of psychosis transition (48). This heterogeneity in recruitment strategies may contribute to a reduction in performance when replicating models in other samples.

Further, the two main frameworks used to describe at-risk populations—the Criteria of Prodromal Syndromes (COPS) (49) and the Comprehensive Assessment of At-Risk Mental State (CAARMS) (50)—may be more or less amenable to these recruitment strategies. As the CAARMS permits for a broader range and duration of APS, a broader recruitment strategy may be warranted. In NAPLS2 (COPS criteria), the sample is younger, has a higher severity of APS at baseline, and experiences a higher psychosis transition rate than the PRONIA study (CAARMS criteria) (22). The CAARMS criteria also tends to be used in countries with socialized medicine (e.g., PRONIA in Europe, the PACE clinic in Australia) whereas the COPS criteria tend to be used in the US, which operates through a privatized healthcare system. Milder cases with more general symptoms may be detected earlier using the CAARMS criteria in countries with significantly reduced barriers to specialized healthcare.

Another notable difference across studies includes the domains of clinical information from which predictor variables are sampled for a given model. Symptom information and certain demographic variables are the most commonly sampled domains included in the models described above, and neurocognitive measures have been included in some, but not all, models. Beyond these domains, other important health-related factors such as substance use (51, 52), sleep patterns (53), and history of traumatic brain injury (54) have been studied in CHR-P samples but rarely included in multivariate prediction models and could differ across cohorts. A more systematic approach across studies to selecting predictors from different variable domains and ensuring the availability of data from these domains could reduce variability in this area as well. In particular, it should be noted that exposure and adherence to pharmacological or psychosocial interventions at baseline may have an association with later transition to psychosis (55, 56); however, medication exposure, medication adherence, and participation in therapy are rarely included in prognostic models. While some descriptive studies have shown that eventual converters and non-converters show no difference in baseline antipsychotic exposure (57), emerging trends suggest that antipsychotic use may predict negative outcomes that may be more pronounced in milder CHR-P cases (57, 58). As antipsychotic medications are typically administered for more severe symptom presentations (2), and administration is difficult to control for in naturalistic studies, more systematic research is needed to clarify and establish the relationships between severity, treatment utilization, and eventual clinical outcomes through prediction studies.

Variance in ascertainment and sample enrichment could be addressed through a clinical staging model involving sample stratification based on the severity of symptom clusters (59). More general and less severe symptoms are categorized as a lower risk stage whereas more severe prodromal symptoms are categorized at a higher risk stage, and interventions would be allocated based on this stratification. More research is needed to validate such a staging model, as baseline symptoms may not predicate linear progression of the psychosis risk syndrome. Poor outcomes could include psychosis transition or worsening symptoms, but may also include the development of other forms of psychopathology. The ability to measure these outcomes will depend on the risk enrichment within the sample, resulting from the ascertainment strategies implemented. To validate prediction models across heterogeneous samples, patient distress, and/or method of ascertainment should be included as a co-variate and future studies should explore the relationships between distress, ascertainment method and level of risk for psychosis.

In examining the most predictive variables included in the prediction models described (e.g., suspiciousness and unusual thought content), it is worth considering the potentially tautological relationship with the definition of psychosis transition (e.g., the presence of psychotic levels of positive symptoms). The question arises of whether the at-risk state is explained by severity of specific positive symptoms which precede eventual transition to psychosis, or whether a more complex risk profile—which may include these symptoms—best explains the at-risk syndrome. In other fields of biomedicine, risk syndromes signal a state during which intervention could successfully delay or prevent the onset of disease. Mild Cognitive Impairment (MCI) is a condition between normal cognitive functioning and impaired cognitive functioning which typically precedes Alzheimer's disease and instantiates the risk state for eventual severe cognitive decline. With proper assessment, early identification and intervention may slow the development of Alzheimer's disease from this precursor state (60). In the clinical high-risk state, positive symptoms seem to be specific to psychosis (2) and thus signal an important opportunity for more intensive assessment and/or intervention regardless of the presence of other risk factors. Nevertheless, the interaction of positive symptoms with other risk factors suggest that these symptoms alone may not be sufficiently predictive of either conversion to psychosis or remission (15), thus reaffirming the ongoing effort to build informative multivariate models that predict heterogeneous outcomes from the CHR-P state that could inform the optimal intervention for a given risk profile.

Another significant methodological challenge in validating and implementing risk models is the necessity of converting a continuous output value to a binary classification output. In the models described, the output is a score ranging from 0.0 to 1.0 indicating the likelihood an individual will experience the outcome of interest (e.g., psychosis transition). The optimal score cutoff that correctly classifies the eventual outcome may differ across studies. In NAPLS2, a score of 0.2 indicated that conversion occurred at a higher rate than non-conversion above the 0.2 predicted risk level (15); however, when tested in the PRONIA sample, the 0.2 cutoff did not yield the same results, and accounting for between-cohort differences in average demographics and symptom severity were necessary to achieve robust external validation at the 0.2 cutpoint (22). These adjustments pose a barrier to clinical implementation and the assessment of new help-seeking individuals and further inquiry into how to calibrate across diverse risk samples will be an essential step toward the clinical implementation of prediction models.

Beyond clinical information, several biomarkers have also been considered for inclusion in the prediction models described. The role of biomarkers in prediction has been reviewed elsewhere (61); thus, we will focus on the additive value of biomarkers to existing clinical models. In this context, no added biomarkers have been shown to replicate across models or studies. In the PRONIA study, a combined clinical and neuroimaging model outperformed either modality alone in predicting social functioning outcomes. This finding, although not yet replicated, suggests that specific configurations of clinical and biological information may achieve the best predictive accuracy as compared to either method independently, consistent with findings that both neuroanatomical and behavioral changes are associated with psychosis transition (62).

Two studies have examined the potential additive role of biomarkers to the NAPLS2 risk calculator. The first added a measure of deviance in neuroanatomical maturity (or “brain age”), previously shown to predict psychosis transition in 12–17 year-olds (63). When added to the risk calculator, however, brain age completely overlapped with chronological age (64), itself a significant predictor of conversion in this sample, with younger cases showing a higher risk. Another study added baseline cortisol to the NAPLS2 risk calculator and found that this measure did contribute significantly to the model's ability to discriminate between eventual converters and non-converters (65). This finding (also not yet replicated) suggests that this measure of baseline cortisol may capture a meaningful biological pattern not otherwise captured through clinical measures.

Further considerations for the inclusion of biomarkers relate to the portability to clinical practice. A major advantage of clinical prediction models is that clinical assessments are inexpensive, non-invasive, and scores can typically be determined within a single day, if not immediately. Thus, clinical decisions could be made quickly. Administering biological tests typically involves a higher cost, longer time to administer or obtain results (e.g., assaying blood or saliva samples, processing MRI scan results), and more invasive or uncomfortable procedures. To optimize clinical utility, biomarkers should contribute significantly to the predictive accuracy of clinical risk models in order to outweigh the higher costs in time, money, and subject burden inherent in obtaining these measures.

As psychosis transition has been the primary outcome of interest in the CHR-P research paradigm, the most substantial progress has been made in predicting this outcome. Other outcomes of interest have received less attention in the field until recently, but warrant further exploration, as it has been understood for some time that the CHR-P population comprises heterogeneous clinical presentations and outcomes (66). The future of risk prediction in the CHR-P paradigm might include the use of a set of algorithms to predict an array of outcomes and the predicted time course of each of these outcomes. This ambitious goal will be discussed more in depth in a latter portion of this paper.

## Prevention

Complementary to the goal of predicting clinical outcomes is the goal of delaying or preventing psychosis through early interventions. A recent review found that only 20 randomized controlled trials (RCTs) testing the effectiveness of either psychosocial (10 of these trials), pharmacological interventions (7 trials), or both (3 trials) had been completed specifically for CHR-P individuals (67). Even within this small number of trials, modalities of treatment and measured outcomes varied greatly. While psychosis transition is a primary outcome, other outcomes include change in positive symptoms, change in negative symptoms, levels of functioning, quality of life and other outcomes related to psychopathology, such as depression and anxiety.

Cognitive behavioral therapy (CBT) has been studied more than other psychosocial modalities (e.g., family interventions, cognitive remediation, and integrative psychotherapy), but has yielded mixed results in either preventing the onset of psychosis or reducing APS. While some studies demonstrated a reduction in rates of psychosis transition (68, 69) or APS reduction (70), others showed no effect of CBT (71) or a larger effect of another modality (e.g., directive listening) in reducing symptom distress (72). Of the other psychosocial modalities, family-focused therapy (FFT) reduced symptoms but not transition rates (73); integrative psychological therapy decreased transition rates at 12 and 24 months (74); and cognitive remediation had no effect on symptoms (75) but improved verbal memory, processing speed and social adjustment (76, 77). Of the pharmacological therapies tested, large trials of omega-3 fatty acids showed no effect in reducing transition rates and symptoms as compared to placebo (78, 79) despite promising earlier studies to this effect (80). Trials of the N-methyl-D-aspartate receptor modulators D-serine and glycine showed no effect in either reducing transition rates or APS (81, 82). Trials with antipsychotics have also been mixed. Antipsychotic medications alone improved positive symptoms but not transition rates (83, 84) and antipsychotics in combination with psychosocial therapy had a more significant effect (85, 86).

Importantly, recent meta-analyses showed that, integrated across studies, no psychosocial or pharmacological interventions significantly reduce APS or transition rates, despite promising results from individual trials (87–90). Further, limitations inherent in both pairwise and network meta-analytic methods render the results of these studies even less conclusive due to comparing unequal control groups across studies, the small number of overall intervention studies, and unequal comparisons across SIPS-based studies and CAARMS-based studies (67). In addition, more recent intervention studies were powered based on higher rates of psychosis transition observed in early CHR-P studies that have since declined, thus resulting in studies that were ultimately underpowered (67).

### Gaps and Future Directions in Prevention

A significant gap in prevention work to date is that the heterogeneity within CHR-P samples has been largely ignored, potentially contributing to the mixed and null results described (12). One way to overcome this is to incorporate some measure of an individual's outcome likelihood into clinical trial design and analysis. A recent study incorporated predicted risk of conversion (15) into re-examining the efficacy of FFT and found that higher risk individuals showed greater APS improvement with the FFT intervention as compared to the control group, but lower risk individuals showed no difference in APS improvement between the two intervention groups (91). A concrete step toward improving our understanding of effective interventions would be to similarly re-examine results of existing trials by incorporating conversion risk as either a measure of stratification or as a covariate in predicting the trial outcome. Another step toward improving the precision of clinical trials would be to incorporate baseline risk assessment and subsequent stratification into the initial design and randomization scheme. These two recommendations represent a crucial way in which the interdigitation of prediction and prevention could broaden the field's scope of inquiry and improve outcomes.

Another way to address outcome heterogeneity is to develop a core outcomes assessment set (COS) for clinical trials. The primary target of interventions would be expanded beyond psychosis transition to include change in positive, negative, anxiety, or depression symptoms, functioning, neurocognition, and recovery or remission (92). Determining a set of outcomes that were included in all trials would facilitate direct comparisons of interventions across trials (93). As prediction models start to extend beyond measuring conversion to psychosis as the primary outcome of interest, so should target outcomes of clinical trials. Whether it is possible to prevent the development of frank psychosis is unknown; however, interventions could likely ameliorate APS and comorbid symptoms of anxiety and depression or improve functioning. Given that the majority of CHR-P individuals do not convert to psychosis yet continue to manifest moderate levels of symptoms and functional impairments (33, 41, 94), targeting non-conversion outcomes is essential to alleviating impairment for this group.

## Future Directions of Inquiry

Ultimately, joining the two tasks of prediction and prevention more systematically could significantly increase the utility and precision of findings from the CHR-P paradigm. Understanding an individual's probable trajectory is most helpful when there is a chance to intervene and potentially improve this trajectory. Approaching this goal could be relatively straightforward [e.g., adding risk scores to clinical trial outcome analysis or stratification (91)], although expanding the scope of inquiry and methods used to interrogate relevant questions will help support the effort to leverage predictive analytics toward improving outcomes.

### Improving Prediction Algorithms for Treatment Selection

Increasingly sophisticated machine learning classification algorithms, such as random forest, support vector machines, Bayesian classifiers, etc., have been used in the CHR population (and psychiatry more broadly) to predict individual outcomes (95). Multi-site consortium studies (e.g., NAPLS, PRONIA, PSYSCAN [http://psyscan.eu]) produce large sample sizes and the opportunity to develop generalizable and robust prediction models with external validation procedures. In these large samples, which are most conducive to implementing machine learning techniques, certain guidelines should inform the implementation of these methods (96). Beyond sample size, the number and type of predictor variables included should be carefully considered. Too many predictors could lead to over-fitting, instability and poor generalization of a model. Thus, feature selection should be performed prior to model fitting either through a data-driven approach, a theory-driven approach, or a hybrid of these two approaches which could leverage the strengths of each (97). The number of features to retain for model development is another key consideration; in general, it is recommended that the ratio of predictors to outcome instances should be approximately 10:1 (98).

A major obstacle faced for prediction in the CHR-P population is the low base rate of conversion to psychosis (99). Machine learning algorithms are biased toward predicting the majority outcome class and accuracy alone cannot provide sufficient information about discrimination between true positive and true negative cases. An algorithm predicting remission in 20/100 CHR-P individuals that is 80% accurate could fail to correctly detect any remission cases but still appear to perform well. Thus, metrics such as sensitivity, specificity, balanced accuracy, and area under the curve (AUC) should also be included. Further, balanced sampling techniques should be considered when severely imbalanced classes threaten the generalizability of a model. One such approach is the synthetic minority oversampling technique (SMOTE) which creates a new data set by oversampling the minority outcome class based on information from neighboring data points, thus reducing bias induced during model learning (100). Balanced sampling approaches should only be used in discovery samples and models should not be validated in synthetically balanced samples. Implementing these sampling approaches has shown to increase model performance in predicting remission (45) and should be considered as a tool to improve prediction in the CHR-P population.

Biomarkers and neuroimaging data may be able to improve prediction algorithms that face a potential limitation in achieving the highest level of predictive accuracy with clinical information alone (61). The inclusion of neurobiological data has not yet yielded these results; however, there is some progress in this direction (36, 65). In a controlled simulation, it was shown that environmental, clinical, neuroimaging and blood biomarker assessments were sequentially examined as testing stages, wherein each subsequent modality added to the positive predictive values (PPV) not detected from the previous modality (59). This type of staged or dynamic approach to risk assessment could greatly increase our ability to predict outcomes, but may become cumbersome in clinical implementation. If overwhelming evidence suggests that the inclusion of a biomarker reliably adds value to individual prediction, it should be included in a prognostic assessment. A decision then arises of whether the field should focus on improving prognostic accuracy by adding biomarkers, or on mobilizing existing prediction efforts with more pragmatic clinical models to inform the selection and development of interventions to improve outcomes more proximally.

A method that warrants more in-depth study involves updating baseline prediction models with clinical information from follow-up visits, called joint modeling (101). This method has been applied in two CHR-P samples and showed significantly better performance than baseline clinical models alone (102, 103). Very few studies have approached risk prediction with heterogeneous clinical profiles and developmental trajectories in mind (33, 38). Ascertaining an individual's probable outcome from a baseline assessment fails to account for variability in syndrome history (e.g., duration of prodromal symptoms prior to initial assessment), and the progression, deterioration, or improvement of symptoms after baseline. Although the baseline “snapshot” approach has been useful to date, a framework that continuously updates based on new or changing clinical information for an individual has the potential to significantly improve outcome prediction.

A related gap in our current research paradigm that warrants expansion is the study of more secondary outcomes such as functioning, remission, and treatment response. This is an area where biomarkers could also help to validate clinical constructs that have not been fully pinned down. Within the example of remission, described previously, the lack of agreement on the time course, duration, and clinical picture of remission could be clarified through careful examination of how symptoms and biomarker signatures co-evolve over time. A very long follow-up period (e.g., > 2 years) would be necessary to understand the time course of remission onset and duration, how remission relates to recovery and relapse, whether neurobiological patterns map onto these clinical presentations, and what would predispose an individual to spontaneous remission as opposed to intervention-driven remission. Similar questions could guide our understanding of outcomes such as social and role functioning as well as neurocognition.

### Improving Treatments and Treatment Selection With Predictive Analytics

The existing literature on psychosocial interventions for the CHR-P population is inconclusive; however, predictive analytics could help account for heterogeneity and inform the type and intensity of recommended services. Biomarkers could potentially play a role in these decisions and more robust inquiry could reveal which mechanisms (e.g., inflammation, neuroplasticity, neurocognition) should be targeted with psychosocial or pharmacological interventions. At this point, mechanisms of these interventions for the CHR-P syndrome are not understood well-enough to match components of an individual's clinical profile to components of effective interventions; however, it would be valuable to have this level of understanding to inform treatment selection.

As no biomarker has consistently replicated in studies assessing individual risk of conversion, the role of clinical predictors is again emphasized. Given that psychosis and its risk syndrome are assessed at the clinical level, symptom-level measurements should also summarize underlying neurobiological changes. With precise clinical measurements, the brain-behavior link is manifested in the individual's responses. Nevertheless, there is room to refine clinical instruments to maximize clinical utility and portability which may also increase consistency in measurements used across consortium studies. Recently, NAPLS investigators developed an abbreviated version of the SIPS—the Mini-SIPS—which could minimize the time and patient burden required to arrive at a relevant risk syndrome diagnosis (104). This measure is yet to be validated although this and similar initiatives could greatly increase the accessibility and transportability of risk assessment measurements in both specialized high-risk clinics and broader healthcare settings.

## Conclusions

Risk prediction and intervention are currently separate but related goals of the clinical high risk for psychosis research paradigm. Progress has been made in ascertaining risk and some progress has been made in identifying effective interventions; however, very little work has combined these tasks—assessing the likelihood of experiencing heterogeneous clinical outcomes and selecting treatment accordingly—to ultimately improve outcomes. The continuation of large multi-site consortium studies will help to facilitate the following ongoing goals: developing and validating risk prediction algorithms for a range of clinical outcomes; identifying time-dependent biomarker signatures that validate clinical trajectories; accounting for sample heterogeneity in clinical trial design; and matching individual clinical profiles to specific mechanisms of effective interventions. Although there is still a lot of work to be done, the future of the CHR-P paradigm holds great promise to significantly improve outcomes for those at the highest levels of risk.

## Author Contributions

MW and TC conceptualized this review. MW conducted the review and drafted the manuscript. TC provided critical revision of the manuscript. All authors approved the final version to be published.

## Conflict of Interest

The authors declare that the research was conducted in the absence of any commercial or financial relationships that could be construed as a potential conflict of interest.

## Publisher's Note

All claims expressed in this article are solely those of the authors and do not necessarily represent those of their affiliated organizations, or those of the publisher, the editors and the reviewers. Any product that may be evaluated in this article, or claim that may be made by its manufacturer, is not guaranteed or endorsed by the publisher.
